# Functional connectivity underlying cognitive and psychiatric symptoms in post-COVID-19 syndrome: is anosognosia a key determinant?

**DOI:** 10.1093/braincomms/fcac057

**Published:** 2022-03-09

**Authors:** Philippe Voruz, Alexandre Cionca, Isabele Jacot de Alcântara, Anthony Nuber-Champier, Gilles Allali, Lamyae Benzakour, Marine Thomasson, Patrice H. Lalive, Karl-Olof Lövblad, Olivia Braillard, Mayssam Nehme, Matteo Coen, Jacques Serratrice, Jérôme Pugin, Idris Guessous, Basile N. Landis, Dan Adler, Alessandra Griffa, Dimitri Van De Ville, Frédéric Assal, Julie A. Péron

**Affiliations:** 1 Clinical and Experimental Neuropsychology Laboratory, Faculty of Psychology, University of Geneva, Geneva, Switzerland; 2 Neurology Department, Geneva University Hospitals, Geneva, Switzerland; 3 Faculty of Medicine, University of Geneva, Geneva, Switzerland; 4 Leenaards Memory Center, Lausanne University Hospital and University of Lausanne, Lausanne, Switzerland; 5 Psychiatry Department, Geneva University Hospitals, Geneva, Switzerland; 6 Diagnostic and Interventional Neuroradiology Department, Geneva University Hospitals, Geneva, Switzerland; 7 Division and Department of Primary Care Medicine, Geneva University Hospitals, Geneva, Switzerland; 8 Internal Medicine Department, Geneva University Hospitals, Geneva, Switzerland; 9 Intensive Care Department, Geneva University Hospitals, Geneva, Switzerland; 10 Rhinology-Olfactology Unit, Otorhinolaryngology Department, Geneva University Hospitals, Geneva, Switzerland; 11 Division of Pulmonary Diseases, Geneva University Hospitals, Geneva, Switzerland; 12 Institute of Bioengineering, Center for Neuroprosthetics, Ecole Polytechnique Fédérale de Lausanne (EPFL), Lausanne, Switzerland

**Keywords:** post-COVID syndrome, anosognosia, neuropsychological deficits, MRI, functional connectivity

## Abstract

Lack of awareness of cognitive impairment (i.e. anosognosia) could be a key factor for distinguishing between neuropsychological post-COVID-19 condition phenotypes. In this context, the 2-fold aim of the present study was to (i) establish the prevalence of anosognosia for memory impairment, according to the severity of the infection in the acute phase and (ii) determine whether anosognosic patients with post-COVID syndrome have a different cognitive and psychiatric profile from nosognosic patients, with associated differences in brain functional connectivity. A battery of neuropsychological, psychiatric, olfactory, dyspnoea, fatigue and quality-of-life tests was administered 227.07 ± 42.69 days post-SARS-CoV-2 infection to 102 patients (mean age: 56.35 years, 65 men, no history of neurological, psychiatric, neuro-oncological or neurodevelopmental disorder prior to infection) who had experienced either a mild (not hospitalized; *n* = 45), moderate (conventional hospitalization; *n* = 34) or severe (hospitalization with intensive care unit stay and mechanical ventilation; *n* = 23) presentation in the acute phase. Patients were first divided into two groups according to the presence or absence of anosognosia for memory deficits (26 anosognosic patients and 76 nosognosic patients). Of these, 49 patients underwent an MRI. Structural images were visually analysed, and statistical intergroup analyses were then performed on behavioural and functional connectivity measures. Only 15.6% of patients who presented mild disease displayed anosognosia for memory dysfunction, compared with 32.4% of patients with moderate presentation and 34.8% of patients with severe disease. Compared with nosognosic patients, those with anosognosia for memory dysfunction performed significantly more poorly on objective cognitive and olfactory measures. By contrast, they gave significantly more positive subjective assessments of their quality of life, psychiatric status and fatigue. Interestingly, the proportion of patients exhibiting a lack of consciousness of olfactory deficits was significantly higher in the anosognosic group. Functional connectivity analyses revealed a significant decrease in connectivity, in the anosognosic group as compared with the nosognosic group, within and between the following networks: the left default mode, the bilateral somatosensory motor, the right executive control, the right salient ventral attention and the bilateral dorsal attention networks, as well as the right Lobules IV and V of the cerebellum. Lack of awareness of cognitive disorders and, to a broader extent, impairment of the self-monitoring brain system, may be a key factor for distinguishing between the clinical phenotypes of post-COVID syndrome with neuropsychological deficits.

## Teaser

Voruz *et al*. demonstrate that a lack of awareness of cognitive impairment (i.e. anosognosia) could be a key factor for distinguishing between different phenotypes of patients with neuropsychological post-COVID-19 conditions.

## Graphical Abstract

**Graphical Abstract:**
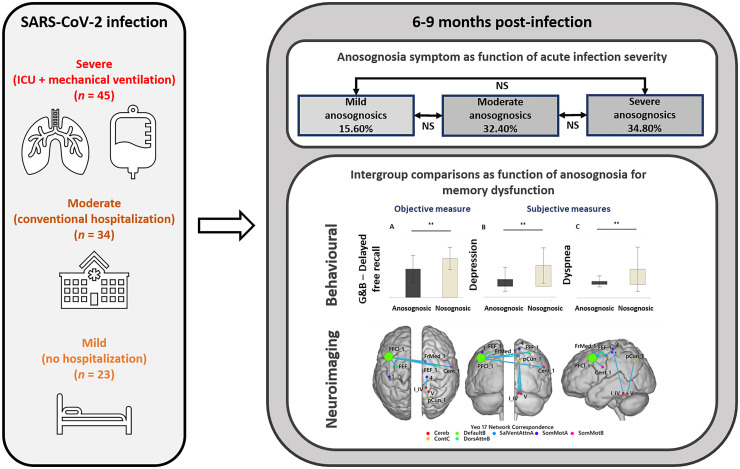

## Introduction

Many studies have demonstrated the presence of both short- and long-term cognitive deficits following SARS-CoV-2 infection^1–5^ Most of them assessed cognition using global efficiency scales (e.g. MoCA, MMSE, M-TICS), mainly in the subacute phase (<12 weeks post-discharge),^1,4^ but some in the chronic phase (>12 weeks post-discharge).^5^ All these studies showed a deterioration in global cognitive efficiency in patients with SARS-CoV-2 (for a review, see Daroische *et al.*^6^). In more comprehensive evaluations (i.e. using specific psychometric tests to evaluate several domains of cognition) impaired memory, executive, attentional and logical reasoning performances were reported.^1–4^ When observing patients with post-COVID syndrome and frequent neuropsychological complaints, clinicians are struck by the frequent lack of awareness of severe cognitive deficits in some patients, as well as by profound subjective neuropsychological complaints in the absence of objective cognitive deficits in other patients. Congruently, one of the above studies that explored the subacute neuropsychological consequences of SARS-CoV-2 infection found that 34% of patients in their sample of post-discharged SARS-CoV-2 patients (with no history of neurological disorders) had post-infection cognitive complaints.^1^ However, when the authors performed comparisons on objectively measured neuropsychological performances between patients with and without subjective cognitive complaints, they failed to find any significant difference. Given that the prevalence of objective cognitive disorders was between 9 and 11.4%, it is tempting to assume that these patients had impaired awareness of their cognitive deficits (i.e. anosognosia), or an over-evaluation of their symptoms. Interestingly, this altered self-monitoring has been observed not only in the subacute phase of COVID-19, but also in the acute phase of the infection,^7^ and not only in patients with the most severe respiratory forms, but also in patients hospitalized with a moderate form of SARS-CoV-2 (present in 9.8% of the hospitalized patients included in this study^7^). Based on this observation, some authors have suggested that anosognosia is a neurological biomarker of the neuroinvasiveness of SARS-CoV-2.^8,9^ More specifically, it has been suggested that when SARS-CoV-2 attacks, by a direct or indirect pathway the CNS, it may trigger or reveal the onset of neurodegenerative pathologies that would otherwise have remained in a prodromal phase for a longer period of time.^10,11^ Neuroimaging studies support the neuroinvasiveness hypothesis (for a review, see Parsons *et al.*^8^ and Manca *et al.*^12^) and more specifically the impact of SARS-CoV-2 on structures involved in self-monitoring. For example, a fluorodeoxyglucose (^18^F) PET study found reduced brain metabolism measures, especially in the olfactory, frontal and limbic systems,^13,14^ which are closely involved in self-awareness disorders, as described below. To date, neuroimaging events have seldom been associated with cognitive measures in SARS-CoV-2. Nevertheless, altered consciousness in the acute phase of the disease, one of the most frequently identified cognitive symptoms, has been associated with various changes in brain structure (e.g. stroke),^12^ although it has also been observed in patients with no visible changes on structural MRI.^15–18^ This points to non-structural brain damage by SARS-CoV-2 and raises the question of functional brain damage. Finally, a meta-analysis of neuroimaging data using linear diffusion-based models of pathological spread in SARS-CoV-2 highlighted a specific reduction in the connectivity of thalamo-cortico-cerebellar pathways, which are known to be involved in self-awareness and arousal.^8^ Accordingly, anosognosia, combined with reduced brain functional connectivity, may be a key factor for distinguishing between different clinical profiles following SARS-CoV-2 infection, differentially impacting cognition and psychiatric deficits, as well as self-reported fatigue, quality of life and olfaction.

*Anosognosia* is defined as an impaired ability to recognize the presence or severity of deficits in sensory, motor, affective and cognitive functioning.^19^ It has previously been described in acute neurological disorders (e.g. stroke) and both autoimmune (e.g. multiple sclerosis) and neurodegenerative (e.g. Alzheimer's disease) diseases. Interestingly, in neurological conditions, anosognosia has been identified as a discriminating factor for the presence and nature of cognitive disorders, psychiatric symptoms and fatigue, as well as for self-reported quality of life.^20,21^ Regarding cognitive symptoms, several studies among patients with mild cognitive disorder or Alzheimer's disease have highlighted significant differences in cognitive performances, with significantly poorer performances among anosognosic versus nosognosic patients for executive functions.^22,23^ Regarding memory functions, the literature is not unanimous, as some studies have failed to find a difference,^24,25^ whereas others have highlighted links between the severity of anosognosia and memory dysfunction.^26,27^ Regarding psychiatric symptoms, studies have also reported significantly lower levels of anxiety and depressive symptoms^28^ in anosognosic patients, but greater apathy,^29^ while anosognosic patients with Alzheimer's disease have better self-reported quality of life than nosognosic patients.^30^ It is important to note that in these studies, psychiatric symptoms and quality of life were self-assessed, and reports may therefore have been biased by anosognosia. Regarding fatigue, one study found that patients with multiple sclerosis were unaware of the severity of their fatigue, despite the fact that it is one of the most prominent symptoms of the disease.^21^ Interestingly, anosmia, one of the most common symptoms following SARS-CoV-2 infection,^31,32^ has been associated with anosognosia in neurodegenerative diseases, with some patients being unaware of their olfactory difficulties.^33^

In the field of self-awareness, several models have been developed.^20^ The cognitive awareness model (CAM)^34,35^ hypothesizes the existence of a metacognitive awareness system. In this system, sensory input passes through the networks involved in episodic memory, before interacting with the networks involved in the storage of personal data and an evaluation system (including a comparison system). On this theoretical basis, several studies have used functional MRI (*f*MRI) to highlight the involvement of the default mode network (DMN) in self-consciousness.^36–38^ The DMN is thought to comprise three functionally dissociable subsystems (dorsomedial prefrontal, medial temporal, midline core).^39^ Recent *f*MRI studies among patients who are anosognosic for memory dysfunction have shown a lack of connectivity between the precentral cortex and orbitofrontal cortex (OFC), and between the OFC and hippocampus,^37^ together with reduced intrinsic connectivity between the posterior inferior parietal cortex, retrosplenial cortex of the ventral precentral cortex and middle temporal gyrus (involved in the dorsomedial DMN subsystem), and reduced connectivity between the hippocampus, ventral precentral cortex and ventromedial prefrontal cortex.^38^ Based on these empirical results, authors have suggested that the dorsomedial prefrontal DMN subsystem includes the CAM's personal data storage module, the medial temporal subsystem includes the episodic memory module and the midline core subsystem is involved in the evaluation and comparison processes.^38^

In this context, by including 102 patients in the chronic phase of post-COVID syndrome (at 6–9 months post-SARS-CoV-2 infection), the primary aim of the present study was to determine the prevalence of anosognosia for memory deficits according to the severity of the infection in the acute phase. Our second aim was to determine whether anosognosic patients with post-COVID syndrome have a different cognitive/psychiatric profile from nosognosic patients, with a different impact on their quality of life, and whether these profiles are mirrored by substantial differences in brain functional connectivity. Based on previous results,^1,8^ and on clinical observations, we assumed that more patients with a moderate or severe form versus a mild form during the acute phase would exhibit anosognosia for memory dysfunction (H1). Based on the results for cognitive performances in neurodegenerative diseases,^22,23,26,27^ we expected anosognosic patients with post-COVID syndrome to exhibit significantly more cognitive impairments than nosognosic patients, notably for executive and memory functioning. Moreover, we expected to observe significantly fewer self-reported psychiatric symptoms, except for apathy,^29^ as well as better self-reported quality of life in anosognosic versus nosognosic patients with post-COVID syndrome (H2).^28,30^ Finally, based on CAM,^34,35^ and on anatomical and functional findings for anosognosia,^37,38^ we expected to observe reduced functional connectivity in the DMN network, as well as in the primary somatosensory^40^ and frontal-attentional networks^41^ in anosognosic versus nosognosic patients (H3).

## Materials and methods

### Participants

We recruited 102 patients who had mild (not hospitalized; *n* = 45), moderate (conventional hospitalization; *n* = 34) or severe (intensive care unit hospitalization and intubation; *n* = 23) disease in the acute phase at 227.07 ± 42.69 days post-SARS-CoV-2 infection (confirmed by a positive polymerase chain reaction test). The interval between infection and testing was 225.00 ± 37.16 days for mild patients, 231.56 ± 50.35 days for moderate patients and 236.87 ± 40.65 days for severe patients, with no significant difference between groups (*P* > 0.34). Participants completed a battery of neuropsychological, psychiatric, fatigue, quality of life, olfactory recognition and dyspnoea tests and questionnaires. The tests were administered by clinical psychologists (mean duration: ∼180 min), and the questionnaires were administered online using Qualtrics software (Qualtrics, Provo, UT, USA) (mean duration: ∼60 min). All participants underwent a full neurological assessment by board-certified neurologists (F.A. and G.A.).

### General procedure

Participants were recruited either via admission lists provided by their treating doctors (M.N., L.B. and O.B.) at Geneva University Hospitals, or from the COVID-COG cohort (F.A. and J.A.P.). For each patient, we carried out a medical file review, followed by a telephone call inviting the patient to take part in the study, if all the eligibility criteria were met. Exclusion criteria were a history of neurological or psychiatric disorders (two of the included participants had an episode of mild depression more than 10 years before their SARS-CoV-2 infection), cancer (to exclude possible chemotherapy- and radiotherapy-related cognitive impairment), neurodevelopmental pathologies, pregnancy and age above 80 years.

### Ethics

After being given a complete description of the study, participants provided their written informed consent. The study was conducted in accordance with the Declaration of Helsinki, and the study protocol was approved by the cantonal ethics committee of Geneva (CER-02186).

### Assessments

#### Objective measures

##### Neuropsychological assessment

Comprehensive and psychometrically validated (for French speakers) tests were conducted for the neuropsychological assessment. For executive functioning, we administered the Stroop task, Trail Making Test and categorical and lexical verbal fluency test from the Groupe de Réflexion sur l’Évaluation des Fonctions Exécutives battery.^42^ Verbal and visuospatial working memory were assessed using the backward digit span^43^ and backward Corsi test.^44^ To assess attentional functions, we measured focused attention, divided attention, phasic alertness, working memory and inhibition using the Test for Attentional Performance.^45^ Regarding memory systems, short-term memory systems were assessed with the forward digit span^43^ and the Corsi test,^44^ verbal episodic memory with the 16-item Grober and Buschke free/cued recall paradigm^46^ and visual episodic memory with the delayed recall of the Rey-Osterrieth Complex Figure test.^47^ For instrumental functions, language was assessed with the BECLA battery,^48^ ideomotor praxis with a short validated battery,^49^ visuoconstructive abilities with the Rey-Osterrieth Complex Figure test^47^ and visuoperceptual functions with four subtests from the Visual Object and Space Perception battery.^50^ Puzzle and Matrices subtests of the Wechsler Adult Intelligence Scale–Fourth Edition (WAIS-IV)^51^ were used to assess logical reasoning. Finally, multimodal emotion recognition was assessed with the Geneva Emotion Recognition Test.^52^

##### Olfaction

Olfactory performance was measured with the Sniffing Sticks test battery. Three thresholds were set^53^: patients with an identification score of 0–7 were considered anosmic, 8–12 hyposmic and 13–16 normosmic.

#### Self-report measures

##### Self-reported acute and long-term somatic symptoms

Patients were given the opportunity to self-report their acute symptoms (retrospectively) and their current symptoms (6–9 months post-infection). Self-reported symptoms were evaluated using an online (Qualtrics) questionnaire, where participants answered *yes*/*no* to the presence of a list of symptoms (runny nose, sore throat, muscle pain, loss of sense of smell, taste disorder, dry cough, productive cough, fever, digestive symptoms, fatigue, difficulty breathing, chest pain, headache, somnolence, non-restorative sleep, insomnia, waking up feeling choked or suffocated, snoring, interruption of breathing during sleep, other, none). They also had the opportunity to describe the presence of other symptoms.

##### Long-term cognitive complaints

Self-reported cognitive complaints were measured with the Cognitive Complaints Questionnaire (QPC),^54^ while executive function complaints were measured with the Behavior Rating Inventory of Executive Function-Adult Version (BRIEF-A).^55^

##### Long-term emotional abilities

Cognitive reappraisal of an emotional episode and expressive emotional suppression abilities were measured with the Emotion Regulation Questionnaire,^56^ and susceptibility to others’ emotions with the Emotion Contagion Scale.^57^

##### Long-term psychiatric symptoms

We administered questionnaires assessing the following psychiatric symptoms: depressive symptoms (Beck Depression Inventory-Second Edition^58^), anxiety (State–Trait Anxiety Inventory^59^), apathy and its distinct subtypes (Apathy Motivation Index^60^), posttraumatic stress disorder (Posttraumatic Stress Disorder Checklist for DSM-5^61^), manic symptoms (Goldberg Mania Inventory^62^), dissociative symptoms in the patient's daily life (Dissociative Experience Scale^63^) and current stress perception (Perceived Stress Scale—14 items^64^).

##### Long-term sleep disorders, insomnia and fatigue

Potential sleeping disorders were assessed with the Insomnia Severity Index,^65^ and symptoms of sleepiness in daily life with the Epworth Sleepiness Scale.^66^ A self-report fatigue questionnaire^54^ probing the multiple dimensions of fatigue (cognitive, physical, social and psychological) was also used in the protocol.

##### Long-term dyspnoea

Dyspnoea was evaluated with a self-report questionnaire^67^ that distinguishes between the physical and affective aspects of self-reported dyspnoea.

##### Long-term quality of life

A self-report questionnaire of quality of life [Short Form 36 Health Survey (SF-36)^68^] was administered. The SF-36 scale makes it possible to measure both the physical and mental aspects of quality of life on a daily basis. The following areas are measured: overall health, physical function, physical role, emotional role, social function, physical pain, emotional wellbeing, vitality score, health modification.

#### Consciousness of disorders: self-appraisal discrepancy

To gauge consciousness of cognitive and olfactory impairments, we calculated self-appraisal discrepancy scores. Objective olfactory scores were categorized as either normal (=1) or impaired (hyposmia and anosmia = 0). Self-reported olfactory ability was categorized as normal (=1) or impaired (=2). We then performed the following subtraction: objective score—self-reported score. Thus, patients with anosognosia for their olfactory functions obtained a score of −1 and patients with nosognosia for their olfactory functions obtained a score of 1. This methodology has been validated elsewhere.^33^

##### Cognition

We first calculated standardized QPC^69^ scores, dividing the raw scores of the self-report questionnaire into four categories: 0 = normal behaviour; 1 = limited influence on daily life; 2 = noticeable influence on daily life and 3 = substantial influence on daily life. Then, each standardized memory score was subtracted from the standardized cognitive complaint score for the relevant function. Self-appraisal discrepancy scores ranged from −3 to 3, with any score below 0 indicating anosognosia.

##### Olfaction

We compared objective scores on the Sniffing Sticks test battery (scores >12 categorized as 1; scores <13 categorized as 0) and patients’ complaints at 6 months (absence of olfactory complaints = 1; presence of olfactory complaints = 0) through a subtraction, such that a score of −1 was equivalent to an absence of consciousness for olfactory disorders.

#### Performance validity measures

The validity of the performances, as well as the presence of non-congruent symptoms, was measured with the BRIEF-A^70^ and the WAIS-IV Digit Span subtest, giving us an opportunity to evaluate non-credible performances and their validity.^71^ The validity analysis revealed a homogeneity of performance in the 102 patients selected for this study. No patients were excluded because of the presence of non-congruent symptoms.

### Image acquisition

A total of 49 participants (mild: *n* = 19; moderate: *n* = 21; severe: *n* = 9) underwent MRI scans at the CIBM Center for Biomedical Imaging in Geneva, on a Siemens Magnetom Prisma Fit 3 T scanner. Of these 49 participants, 11 were anosognosic and 38 nosognosic. Analysis revealed no significant differences between the anosognosic and nosognosic groups on age (*P* = 0.061), sociocultural level (*P* = 0.923), sex (*P* = 0.861) or handedness (Supplementary Table 1). Intergroup analysis also failed to reveal any significant differences between anosognosic and nosognosic patients on either the interval between infection and MRI (anosognosic: 287.08 ± 38.30 days; nosognosic: 265.54 ± 51.07 days; *P* = 0.146) or the interval between neuropsychological testing and MRI (anosognosic: 49.90 ± 26.95 days; nosognosic: 33.68 ± 23.93 days; *P* = 0.078) (Supplementary Table 2). Data from five of them were excluded (high movement and poor registration). Structural images were obtained with a T_1_-weighted (T_1_w) magnetization-prepared rapid acquisition gradient-echo sequence with an isotropic voxel size of 0.9375 × 0.9375 × 0.9 mm (Supplementary Table 3). Resting-state functional images were acquired through a multiband accelerated echo-planar sequence with an isotropic voxel size of 2.5 mm^3^, 64 slices, repetition time of 1 s for a total of 7 min 59 s of acquisition time (480 volumes; Supplementary Table 4).

We report the results of the preprocessing performed using *f*MRIPrep 20.2.3,^72,73^ which is based on Nipype 1.6.1.^74^

#### Anatomical preprocessing

Each T_1_w volume was corrected for intensity non-uniformity using *N4BiasFieldCorrection* v2.1.0^75^ and skull-stripped using *antsBrainExtraction.sh* v2.1.0 (using the OASIS template). Spatial normalization to the ICBM 152 Non-linear Asymmetrical template version 2009c^76^ was performed through non-linear registration with the *antsRegistration* tool of Advanced Normalization Tools (ANTs) v2.1.0,^77^ using brain-extracted versions of both T_1_w volume and template. Brain tissue segmentation of CSF, white matter (WM) and gray matter was performed on the brain-extracted T_1_w using *fast*^78^ (FSL v5.0.9).

#### Functional preprocessing

Functional data were slice time corrected using *3dTshift* from AFNI v16.2.07^79^ and motion corrected using *mcflirt* (FSL v5.0.9^80^). This was followed by co-registration to the corresponding T_1_w using boundary-based registration^81^ with six degrees of freedom, using *flirt* (FSL). Motion correcting transformations, BOLD-to-T_1_w transformation and T_1_w-to-template (MNI) warp were concatenated and applied in a single step using *antsApplyTransforms* (ANTs v2.1.0) using Lanczos interpolation. Physiological noise regressors were extracted applying component-based noise correction (CompCor).^82^ Principal components were estimated for the two CompCor variants: temporal component-based noise correction (tCompCor) and anatomical component-based noise correction (aCompCor). A mask to exclude signal with cortical origin was obtained by eroding the brain mask, ensuring it only contained subcortical structures. Six tCompCor components were then calculated including only the top 5% variable voxels within that subcortical mask. For aCompCor, six components were calculated within the intersection of the subcortical mask and the union of CSF and WM masks calculated in T_1_w space, after their projection to the native space of each functional run. Frame-wise displacement^83^ was calculated for each functional run using the implementation of Nipype and volumes with a frame-wise displacement >0.7 mm were excluded (Supplementary Table 5). Many internal operations of FMRIPREP use Nilearn,^84^ principally within the BOLD-processing workflow. For more details of the pipeline, see the section corresponding to workflows in the *f*MRIPrep documentation.

### Behavioural statistical analysis

First, patients were divided into two groups according to their anosognosia for memory dysfunction score, regardless of the severity of their respiratory symptoms in the acute phase, by calculating a self-appraisal discrepancy score (anosognosic patients, cut-off <0).^85–87^ To determine whether there was a difference in the proportions of self-reported symptoms between anosognosic and nosognosic patients for the acute and long-term phases, and given that the data were distributed in a categorical manner, we ran *χ*^2^ comparison analyses. Mann—Whitney tests with false discovery rate (FDR) correction on neuropsychological scores, psychiatric symptoms, fatigue, quality of life, dyspnoea and olfactory abilities were performed to compare the performances of the subgroups. Dichotomous data (sociodemographic variables such as age and sociocultural level; acute complaints and complaints at 6 months; anosognosia and nosognosia for each severity group) were analysed using *χ*^2^ tests. All results were FDR corrected (*P* < 0.050).

### Structural MRI inspection

First, the neuroimaging data were visually analysed to look for noticeable brain lesions such as microbleeds (susceptibility-weighted imaging) and WM damage (fluid-attenuated inversion recovery). Groups (Supplementary Fig. 1) were compared on the total number of microbleeds and impact on WM, with the Wahlund scale.^88^

### fMRI statistical analysis

The processed functional timecourses were averaged into 156 regions of interest (100 cortical regions,^89^ 34 cerebellar regions^90^ and 22 regions from the basal ganglia^91^), and the functional connectivity between pairs of regions was defined with Pearson correlation coefficients. Measures of functional connectivity were converted into *z*-scores with the Fisher *z*-transformation and compared using two-sample *t*-tests to investigate differences between groups. *P*-values were corrected for multiple comparisons with the FDR method.^92^

### Data availability

Non-sensitive COVID-COG data will be made available at the end of the project in open access on a dedicated platform.

## Results

### Prevalence of anosognosia according to the severity of respiratory symptoms in acute phase

Based on anosognosia for memory dysfunction scores, we divided the sample into one group (*n* = 26) of anosognosic patients (*n* = 7 mild, *n* = 11 moderate and *n* = 8 severe) and one group (*n* = 76) of nosognosic patients (*n* = 38 mild, *n* = 23 moderate and *n* = 15 severe). No significant statistical differences were observed between the two groups on age, sociocultural level, handedness, sex, interval between infection and testing or clinical variables (except for chronic renal failure) (see Table 1). We observed that 15.6% of patients who presented mild disease displayed anosognosia for memory dysfunction, compared with 32.4% of patients with moderate disease and 34.8% of patients with severe disease. No significant differences were observed between the moderate and severe groups (*χ*^2^ = 0.849), but trends towards significance were observed between the mild and moderate (*χ*^2^ = 0.078), and mild and severe (*χ*^2^ = 0.070) groups.

**Table 1:** Sociodemographic data of anosognosic and nosognosic patients with COVID-19

	Anosognosic (*n* = 26)	Nosognosic (*n* = 76)	*P*-value
Mean age in years (±SD)	56.58 (±13.12)	56.49 (±9.60)	0.871
Mean education level (±SD)	2.62 (±0.64)	2.68 (±0.50)	0.821
Sex (F/M)	7/19	30/46	0.251
Mean days of hospitalization (±SD)	25.67 (±27.22)	18.27 (±21.90)	0.900
Mean days between infection and testing (±SD)	221.81 (±39.86)	230.25 (±43.83)	0.438
Diabetes in %	19.30	6.60	0.083
Smoking in %	0	10.50	0.085
History of respiratory disorders in %	7.70	17.10	0.242
History of cardiovascular disorders in %	23.10	14.50	0.310
History of neurological disorders in %	0	0	–
History of psychiatric disorders in %	0	2.65^a^	0.402
History of cancer in %	0	0	–
History of severe immunosuppression in %	0	0	–
History of developmental disorders in %	0	0	–
Chronic renal failure in %	7.70	0	0.015*
Sleep apnoea syndrome in %	6.30	14.50	0.147
History of severe immunosuppression in %	0	0	–

^a^ Two patients reported a depressive episode that occurred more than 10 years before infection and which was treated at the time.

* *P* < 0.05.

### Distinct cognitive profiles according to the presence of anosognosia for memory dysfunction

#### Objective measures

##### Neuropsychological deficits

**Figure 1:**
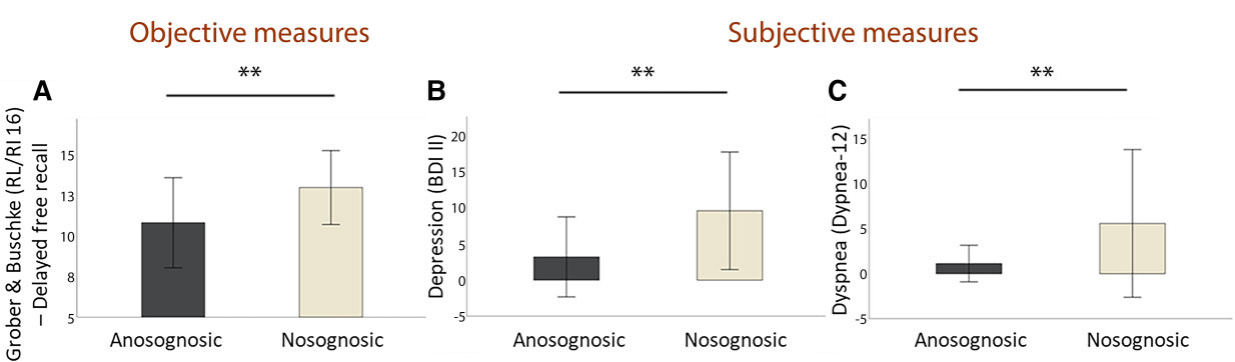
**Objective and subjective (self-report) measures (Mann–Whitney U-tests with FDR correction).** (**A**) Anosognosic patients performed significantly more poorly than nosognosic patients on long-term verbal episodic memory (*z* = 3.37, *P* < 0.001); (**B**) Anosognosic patients reported significantly fewer depressive symptoms than nosognosic patients (*z* = 5.16, *P* < 0.001); (**C**) Self-reported anosognosic patients had significantly less dyspnoea than nosognosic patients (*z* = 3.21, *P* = 0.001).

Analyses with FDR correction revealed a significant difference between anosognosic and nosognosic patients on long-term verbal episodic memory, suggesting reduced verbal memory performance in post-COVID syndrome patients with anosognosia (see Fig. 1). Whereas without passing the FDR significance level, poorer performances were also observed for anosognosic versus nosognosic patients on Rey figure copy time, short-term verbal memory, mental flexibility, phasic alertness and sustained attention, as well as on semantic image matching. No other results were significant (see Table 2).

**Table 2:** Neuropsychological performances (mean ± SD) and significant differences between groups

		Anosognosics (*n* = 26)	Nosognosics (*n* = 76)	*P*-value
Memory				
Verbal episodic memory	Grober and Buschke (FR/CR 16)—immediate recall	15.53 (±0.76)	15.87 (±0.47)	**0.003** **
	Grober and Buschke (FR/CR 16)—sum of three free recalls	28.50 (±6.59)	32.37 (±6.18)	**0.008** *
	Grober and Buschke (FR/CR 16)—sum of three total recalls	44.00 (±3.45)	46.05 (±2.81)	**<0.001** **
	Grober and Buschke (FR/CR 16)—delayed free recall	10.81 (±2.77)	12.96 (±2.25)	**0.001** **
	Grober and Buschke (FR/CR 16)—delayed total recall	14.88 (±1.56)	15.75 (±0.61)	**0.001** **
Visuospatial episodic memory	Rey figure—copy time	195.58 (±81.59)	151.58 (±53.10)	**0.013** *
	Rey figure—score	33.96 (±3.00)	34.15 (±2.94)	0.768
	Rey figure—immediate recall (3′)	17.10 (±6.77)	19.43 (±6.30)	0.246
	Rey figure—delayed recall (20′)	21.92 (±7.74)	24.22 (±6.53)	0.259
Verbal short-term memory	MEM III—Spans	8.27 (±2.23)	9.53 (±2.20)	**0.016** *
Visuospatial short-term memory	WAIS-IV—Spans	8.12 (±2.07)	8.17 (±2.16)	0.296
Executive functions				
Inhibition	Stroop (GREFEX)—interference—time^a^	122.04 (±26.31)	118.56 (±28.46)	0.421
	Stroop (GREFEX)—interference—errors^a^	0.87 (±1.39)	0.04 (±0.20)	0.618
	Stroop (GREFEX)—interference/naming—score^a^	51.43 (±19.47)	49.93 (±22.01)	0.744
Working memory	MEM III—verbal working memory	8.12 (±2.07)	8.38 (±1.87)	0.602
	WAIS-IV—visuospatial working memory	7.50 (±1.73)	7.72 (±1.82)	0.908
	TAP—working memory item omissions	2.38 (±3.02)	2.25 (±2.03)	0.558
	TAP—working memory false alarms	3.85 (±6.04)	3.04 (±3.31)	0.704
Mental flexibility	TMT B-A (GREFEX) —score	77.46 (±75.42)	49.72 (±37.62)	**0.049** *
	Verbal fluency (GREFEX)—literal (2′)	19.54 (±6.67)	22.07 (±6.65)	0.162
	Verbal fluency (GREFEX)—categorical (2′)	28.73 (±10.57)	31.70 (±8.82)	0.120
Incompatibility	TAP—compatibility—false alarms	2.23 (±4.80)	1.64 (±4.64)	0.768
	TAP—incompatibility—false alarms	2.23 (±4.80)	3.25 (±5.61)	0.355
Interhemispheric transfer	TAP—incompatibility—visual field score	1.83 (±2.28)	1.68 (±1.98)	0.937
	TAP—incompatibility task—hands score	2.63 (±4.60)	2.70 (±2.99)	0.396
Attentional functions				
Phasic alertness	TAP—without warning sound—reaction time^b^	250.96 (±32.53)	270.53 (±64.86)	0.392
	TAP—without warning sound—SD of reaction time^b^	36.85 (±17.30)	50.34 (±32.10)	**0.021** *
	TAP—with warning sound—reaction time^b^	253.35 (±33.12)	264.11 (±53.38)	0.626
	TAP—with warning sound—SD of reaction time^b^	39.23 (±14.48)	43.24 (±21.71)	0.640
Sustained attention	TAP—item omissions	13.36 (±9.88)	11.64 (±9.77)	0.314
	TAP—false alarm	18.84 (±27.93)	6.29 (±9.43)	**0.012** *
Divided attention	TAP—total omissions	1.96 (±2.37)	1.84 (±1.99)	0.948
	TAP—total false alarms	1.88 (±2.55)	0.84 (±1.41)	0.052
Instrumental functions				
Language	BECLA—semantic image matching	19.46 (±0.81)	19.80 (±0.54)	**0.009** *
	BECLA—semantic word matching	19.62 (±0.80)	19.78 (±0.51)	0.651
	BECLA—object and action image naming	19.15 (±1.46)	19.43 (±0.84)	0.854
	BECLA—word repetition	14.96 (±0.20)	14.97 (±0.16)	0.930
	BECLA—nonword repetition	9.62 (±0.64)	9.86 (±0.45)	0.357
Ideomotor praxis	Evaluation of ideomotor praxis—symbolic gestures	4.92 (±0.27)	4.87 (±0.44)	0.827
	Evaluation of ideomotor praxis—action pantomimes	9.62 (±0.64)	9.38 (±0.92)	0.463
	Evaluation of ideomotor praxis—meaningless gestures	7.81 (±0.57)	7.79 (±0.62)	0.917
Object perception	VOSP—fragmented letters	18.73 (±2.49)	19.43 (±0.66)	0.104
	VOSP—object decision	16.77 (±3.81)	17.36 (±1.82)	0.866
Spatial perception	VOSP—number localization	8.77 (±2.10)	9.30 (±1.02)	0.936
	VOSP—cubic counting	7.81 (±0.57)	9.50 (±1.01)	0.144
Logical reasoning	WAIS-IV—puzzle	12.92 (±5.04)	13.93 (±5.19)	0.422
	WAIS-IV—matrix	14.42 (±5.48)	16.34 (±4.71)	0.074
Emotion recognition	GERT—emotion recognition task	21.15 (±7.22)	24.09 (±5.91)	0.103

In bold all significative results before and after FDR correction. GERT, Geneva Emotion Recognition Task; TAP, Test of Attentional Performance.

^a^ Data missing for three nosognosic participants owing to colour blindness.

^b^ Data missing for one nosognosic participant.

* *P* < 0.05.

** *P* < 0.05 FDR corrected.

##### Olfaction

Results revealed no significant difference between anosognosic and nosognosic patients on objectively measured olfactory recognition.

#### Self-reported symptoms

##### Neuropsychiatric symptoms

**Figure 2:**
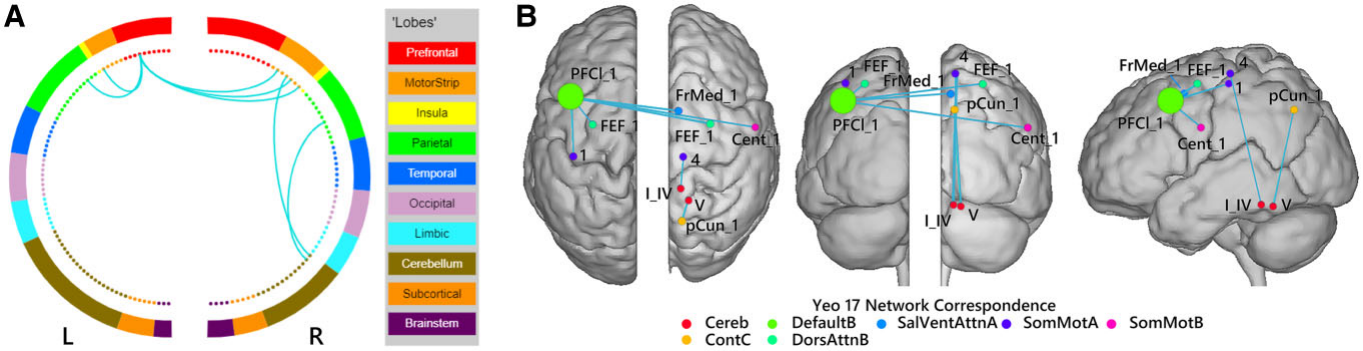
**Patterns of significantly lower functional connectivity in patients with anosognosia for memory dysfunction than in nosognosic patients.** (**A** and **B)** Differences in functional connectivity shown between brain structures (**A**) and in a network representation on a glass brain. Blue links indicate a decrease in the connectivity measurement (mean decrease = −0.3), and node size corresponds to number of connections. Statistical significance was FDR corrected for multiple comparisons (*P* < 0.05, FDR). Networks: Cereb, cerebellum; ContC, control C; DefaultB, default mode B; DorsAttnB, dorsal attention B; SalVentAttA, salience ventral attention A; SomMotA, somatosensory motor A; SomMotB, somatosensory B. Regions: Cent, central sulcus; FEF, frontal eye field; FrMed, frontal medial cortex; pCun, precuneus; PFCl, lateral prefrontal cortex; I_IV and V, Lobules I_IV and V of the cerebellum. Figures were created with BioImage Suite (https://bioimagesuiteweb.github.io/webapp/index.html).

FDR corrected analyses revealed significantly fewer self-reported psychiatric symptoms in anosognosic versus nosognosic patients for depression (see Fig. 1), anxiety and stress perception (see Table 3). Whereas, behavioural apathy, social apathy, posttraumatic stress disorder, dissociative disorder, somnolence and insomnia did not pass the FDR correction. No other results were significant (see Table 3 and Fig. 2).

**Table 3:** Psychiatric symptoms, fatigue, dyspnoea and olfaction (mean ± SD) and significant differences between groups

	Anosognosics (*n* = 26)	Nosognosics (*n* = 76)	*P*-value
Depression (BDI-II)	3.23 (±5.55)	9.47 (±8.10)	**<0.001** **
State anxiety (STAI-state)	25.67 (±7.64)	35.75 (±12.62)	**<0.001** **
Trait anxiety (STAI-trait)	28.96 (±7.97)	35.93 (±11.66)	**0.003** **
Mania (Goldberg Inventory)	14.46 (±9.58)	15.71 (±8.56)	0.519
Apathy (AMI-total)	25.27 (±7.86)	27.78 (±7.86)	0.208
Behavioural apathy (AMI-behavioural)	5.81 (±3.96)	8.20 (±4.39)	**0.014** *
Social apathy (AMI-social)	7.92 (±4.43)	10.41 (±4.36)	**0.013** *
Emotional apathy (AMI-emotional)	11.54 (±4.45)	9.17 (±3.69)	0.030
Posttraumatic stress disorder (PCL-5)	10.00 (±10.19)	17.49 (±14.12)	**0.009** *
Stress (PSS-14)	11.92 (±7.17)	20.41 (±10.62)	**<0.001** **
Dissociative disorder (DES)	4.46 (±4.74)	7.68 (±9.18)	**0.024** *
Emotional Contagion Scale (ECS)	39.69 (±8.38)	40.84 (±6.17)	0.664
Emotional Regulation Questionnaire (ERQ)	40.35 (±12.97)	39.86 (±10.14)	0.942
Somnolence (Epworth)	6.65 (±3.97)	9.42 (±4.31)	**0.005** *
Insomnia (ISI)	5.00 (±2.48)	10.64 (±4.82)	**<0.001** **
Fatigue—total (EMIF-SEP)	37.24 (±12.70)	53.84 (±16.41)	**<0.001** **
Fatigue—cognitive (EMIF-SEP)	36.06 (±13.02)	54.14 (±19.16)	**<0.001** **
Fatigue—physical (EMIF-SEP)	40.38 (±16.80)	60.17 (±19.70)	**<0.001** **
Fatigue—social (EMIF-SEP)	38.61 (±13.29)	52.18 (±16.65)	**0.001** **
Fatigue—psychological (EMIF-SEP)	34.86 (±12.65)	51.40 (±20.50)	**0.001** **
Dyspnoea—total (Dyspnoea-12)	1.13 (±2.03)	5.45 (±8.12)	**0.002** **
Dyspnoea—physical (Dyspnoea-12)	1.00 (±1.56)	3.92 (±4.80)	**0.002** **
Dyspnoea—affective (Dyspnoea-12)	0.13 (±0.61)	1.53 (±3.99)	0.093
Sniff test (anosmia)	12.11 (±2.42)	12.19 (±2.25)	0.259

In bold all significative results before and after FDR correction. DES, Dissociative Experience Scale; EMIF-SEP, Echelle Modifiée d’Impact de la Fatigue; PCL-5, Posttraumatic Stress Disorder Checklist for DSM-5; PSS-14, Perceived Stress Scale—14 items; STAI-S, State Anxiety Inventory; STAI-T,  Trait Anxiety Inventory.

* *P* < 0.05.

** *P* < 0.05 FDR corrected.

##### Fatigue

Analyses revealed significantly less self-reported fatigue in anosognosic versus nosognosic patients (*P* < 0.001), regardless of the subdomain that was measured (cognitive, physical, social or psychological) (see Table 3).

##### Dyspnoea

There were significantly lower scores for total dyspnoea, as well as for the physical domain of dyspnoea in anosognosic versus nosognosic patients (*P* = 0.002 and *P* = 0.002), whereas no significant difference was observed on the affective domain of dyspnoea (*P* = 0.098) (see Table 3 and Fig. 1).

##### Self-reported olfactory abilities

A significantly higher prevalence of unconsciousness of olfactory recognition disorders in anosognosic versus nosognosic patients was found (*χ*^2^ = 0.043).

##### Quality of life

All facets of perceived quality of life were significantly better among anosognosic versus nosognosic patients after FDR correction, except for physical pain (*P* = 0.008) (see Table 4).

**Table 4:** Quality of life (SF-36) (mean ± SD) and significant differences between groups

	Anosognosics (*n* = 26)	Nosognosics (*n* = 76)	*P*-value
Overall health	77.12 (±19.86)	62.30 (±19.96)	**<0.001** **
Physical function	91.73 (±13.63)	78.88 (±20.73)	**0.001** **
Physical role	92.31 (±20.94)	57.89 (±39.20)	**<0.001** **
Emotional role	98.72 (±6.53)	71.06 (±37.85)	**0.001** **
Social function	90.87 (±16.79)	71.55 (±26.35)	**<0.001** **
Physical pain	85.19 (±15.57)	69.84 (±25.32)	**0.008** *
Emotional wellbeing	83.23 (±15.55)	67.68 (±20.77)	**<0.001** **
Vitality score	67.69 (±17.51)	48.36 (±20.71)	**<0.001** **
Health modification	51.92 8 (±14.01)	33.88 (±20.70)	**<0.001** **

For all measure, a higher score indicates a better quality of life. In bold all significative results before and after FDR correction.

* *P* < 0.05.

** *P* < 0.05 FDR corrected.

##### Other symptoms

Anosognosic patients in the chronic phase retrospectively reported fewer sense of smell symptoms in the acute phase than nosognosic patients did, and continued to report significantly fewer symptoms of fatigue at the time of the assessment, compared with nosognosic patients (see Table 5).

**Table 5:** Percentages of self-reported symptoms in the acute phase and 6–9 months post-infection for the two groups

	Anosognosic (*n* = 26)	Nosognosic (*n* = 76)		Anosognosic (*n* = 26)	Nosognosic (*n* = 76)	
	Acute	Acute	*P*-value	6–9 months post	6–9 months post	*P-*value
Runny nose	15.4	31.6	0.110	3.8	5.3	0.773
Sore throat	19.2	17.1	0.806	0	1.3	0.557
Muscle pain	53.8	53.8	0.993	7.7	10.5	0.675
Sense of smell	26.9	53.9	**0.017** **	3.8	5.3	0.773
Taste disorder	34.6	48.7	0.217	6.25	17.02	0.288
Dry cough	53.8	55.3	0.900	0	1.3	0.557
Productive cough	0	9.2	0.109	–	–	–
Fever	61.5	69.7	0.441	–	–	–
Digestive symptoms^a^	42.3	35.5	0.537	3.8	2.6	0.752
Fatigue	76.9	85.5	0.310	23.1	53.9	**0.006** **
Difficulty breathing	42.3	44.7	0.830	0	13.2	0.051
Chest pain	19.2	27.6	0.396	0	2.6	0.403
Headache	50.0	65.8	0.153	7.7	13.2	0.455
Somnolence	15.4	32.9	0.088	3.8	9.2	0.380
Non-restorative sleep	23.1	36.8	0.199	3.8	11.8	0.237
Insomnia	15.4	9.2	0.381	0	18.4	**0.018** *
Waking up feeling choked or suffocated	12.50	10.64	0.838	0	2.6	0.403
Snoring	0	1.3	0.557	0	2.6	0.403
Interruption of breathing during sleep	3.8	5.3	0.985	0	3.9	0.304
Other	23.1	21.1	0.828	7.7	27.60	**0.036** *
None	11.5	1.3	**0.020** **	65.4	17.1	**<0.001** **

In bold all significative results before and after FDR correction.

^a^ Nausea/diarrhea/abdominal pain/inappetence.

* *P* < 0.05.

** *P* < 0.05 FDR corrected.

### Neuroimaging

**Figure 3:**
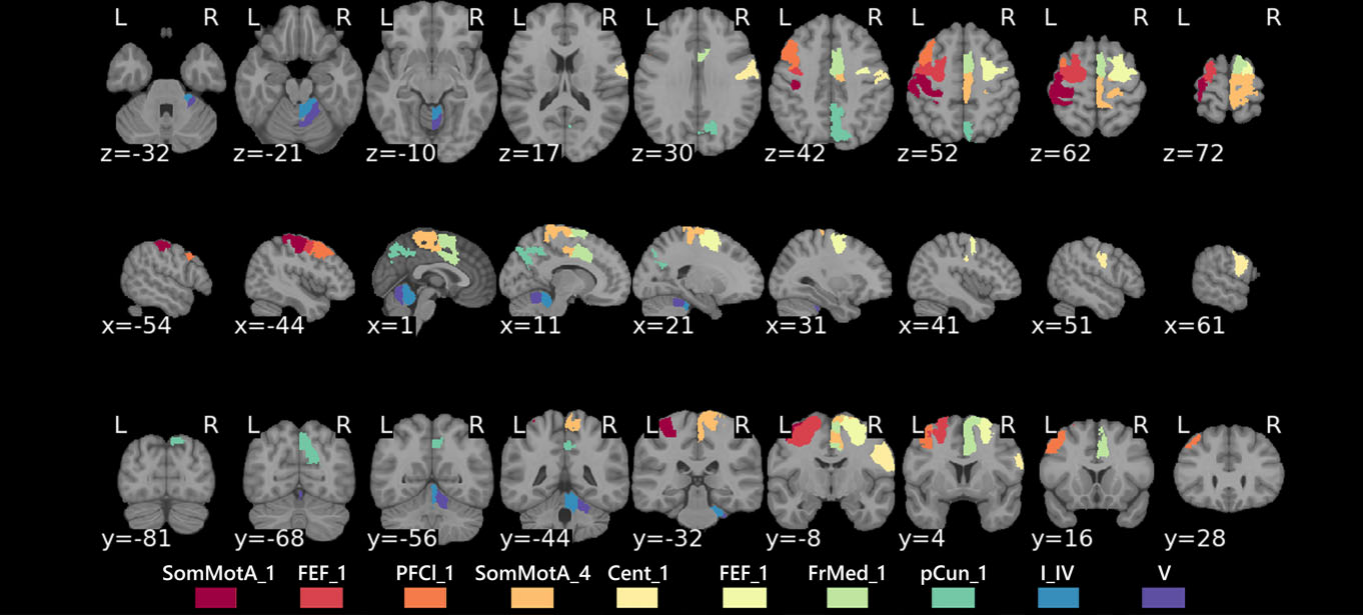
**Anatomical map of affected regions in patients with anosognosia for memory dysfunction.** Regions: Cent, central sulcus; FEF, frontal eye field; FrMed, frontal medial cortex; pCun, precuneus; PFCl, lateral prefrontal cortex; SomMotA, somatosensory motor A; I_IV and V, Lobules I_IV and V of the cerebellum.

First, no difference emerged from the comparisons of structural images (susceptibility-weighted images, FLAIR) (Figs 2 and 3).

Second, our results revealed three patterns of hypoconnectivity in anosognosic versus nosognosic patients: (i) a weaker connectivity (*P* < 0.05 with FDR correction) between the left lateral prefrontal cortex (PFCl) in the default mode network B (DMN_B_) with the following subregions and associated networks: a subregion of the left somatosensory motor network A (SomMot_A_), a subregion of the right somatosensory motor network B (SomMot_B_), the right frontal medial subregion of the salient ventral attention network A (SalVentAttn_A_) and with the bilateral frontal eye field (FEF) in the dorsal attention network B (DAN_B_); (ii) weaker connectivity (*P* < 0.05 with FDR correction) between a subregion of the right SomMot_A_ and the right Lobule I_IV of the cerebellum; (iii) weaker connectivity between the right precuneus in the control network C (CN_C_) and the right Lobule V of the cerebellum (I_V). Overall, the statistical group analysis revealed hypoconnectivity patterns in anosognosic versus nosognosic patients, but these patterns did not survive the FDR correction (uncorrected *P* < 0.01; Supplementary Fig. 2).

## Discussion

The present results demonstrate that anosognosic versus nosognosic SARS-CoV-2 patients, 6–9 months following infection, have (i) greater impairment of memory, (ii) fewer self-reported psychiatric symptoms of depression, anxiety and stress, (iii) better self-reported quality of life and (iv) reduced connectivity between the DMN_B_ (including dorsal and lateral prefrontal cortices), CN_C_, bilateral SomMot_A_, SomMot_B_, SalVentAttn_A_, bilateral DAN_B_ networks (including FEF) and the right Lobules IV and V of the cerebellum. Accordingly, we suggest that anosognosia for memory dysfunction could be used to differentiate between distinct clinical phenotypes of neuropsychological post-COVID syndrome.^85^ Moreover, this marker does not entirely depend on the severity of respiratory symptoms in the acute phase, as it can also affect people who had a mild form (∼15% of the mild sample). This point was later confirmed by prevalence comparison analyses suggesting that there was no significant difference in the prevalence of anosognosia for memory dysfunction according to the severity of acute respiratory symptoms. These findings are in line with cognitive/psychiatric observations of anosognosia in Alzheimer's disease,^20^ pointing to a potentially specific neurological manifestation of SARS-CoV-2. Moreover, our results seem to corroborate previous clinical observations that patients with the most complaints have more severe self-reported anxiety and depressive symptoms scores than those with no complaints,^1^ but a minor impact from a cognitive point of view.^93^ Finally, our neuroimaging results showed a general pattern of reduced connectivity in patients with anosognosia, especially between the left DMN_B_, right CN_C_, as well as bilateral SomMot_A_, SomMot_B_ and bilateral DAN_B_, pointing to a specific neurological impairment in this phenotype following SARS-CoV-2 infection, with no significant difference in structural damage between groups (SI 6). Similarly, lack of awareness (as well as unspecified cognitive decline) was previously observed in patients with COVID-19 who had no structural alterations on MRI.^16–19^ Moreover, the reduced connectivity in the DMN_B_ in our anosognosic patients (in the left PFCl) was also found in a previous neuroimaging study of anosognosia,^38,39^ in which these networks were associated with CAM evaluation and comparison systems.^39^ Moreover, according to CAM, the patterns of reduced connectivity between the Somatomotor and DMN_B_ networks in our anosognosic patients may reflect impaired sensorimotor processing of stimuli, affecting the multimodal processing of cognition. Our results also revealed hypoconnectivity patterns in the somatosensory and SalVentAttn_A_ subregions, including the insula. The involvement of this structure (and the connectivity networks associated with it) has been largely demonstrated in self-awareness and anosognosia for memory impairments (for a review, see Hallam *et al.*^94^). According to CAM,^95^ these results may indicate an impaired switching mechanism between the DMNs and the central-executive control network, inducing anosognosia for memory impairment.^95^ We observed hypoconnectivity patterns in regions of the right cerebellum (Lobules IV and V). The presence of ACE-2 receptors in this structure may account for this effect^96^ explaining the vulnerability of the cerebellum to SARS-CoV-2 damage,^97^ and perhaps the involvement of cerebellar networks in self-awareness.^98^ In terms of neuropsychological deficits observed in anosognosic patients, the reduced memory performances could be linked to connectivity patterns of dorsal regions (including the dorsolateral prefrontal cortex), which have been associated with salience processing (familiarity).^99^ Finally, DMNs, including DMN_B_, have been associated with both encoding and recollection abilities.^99^ Thus, the comparison of neuroimaging data across groups, associated with differential neuropsychological profiles observed in anosognosic patients, are consistent with previous studies^99–101^ (for a review, see Hallam *et al.*^94^). Our results suggest that SARS-CoV-2 has a long-term effect on the CNS and associated cognitive functions.

Lack of awareness or anosognosia seems to concern not only cognitive deficits, but also olfactory abilities. Our results revealed that a greater proportion of anosognosic patients lacked awareness of their olfactory disorders. Thus, infection with SARS-CoV-2 probably affects awareness of body signals in general through an alteration of the somatosensory system that is central to embodiment,^102^ preventing anosognosic patients from correctly perceiving bodily cues. Interestingly, this may already occur during the acute phase, as our anosognosic patients retrospectively reported significantly fewer physiological olfactory complaints during the acute phase than nosognosic patients did. This may explain so-called *happy hypoxia* in SARS-CoV-2 which would be due to a lack of conscious awareness, or anosognosia, of severe respiratory failure.^103,104^ Our data also seemed to highlight a greater long-term lack of awareness of olfactory and respiratory deficits among anosognosic patients, as attested by self-reported olfactory complaints and dyspnoea. Self-reported fatigue and quality of life also appeared to be determined by this factor, with very low perceived fatigue and very good quality of life in anosognosic patients, whereas cohort data in patients with SARS-CoV-2 suggest that chronic fatigue and reduced quality of life are among the most prominent symptoms of post-COVID syndrome.^105^ Being unaware of their difficulties, these patients may not feel the effects of their cognitive and physical symptoms on a daily basis.

Thus, our cognitive, psychiatric, olfactory, dyspnoea, fatigue, quality of life and brain connectivity results point to an impairment of global self-awareness, which may be related to a specific clinical phenotype of neurological post-COVID syndrome. We speculate that these symptoms may already be present in the acute phase and persist over the long term because of neurological damage. The question about the possible origin of this lack of awareness or self-monitoring remains unanswered, and multidetermined models of anosognosia/self-monitoring including systems (e.g. respiratory system) other than just the CNS will have to be developed in order to understand this phenomenon,^35^ especially in the case of post-COVID syndrome. On the basis of the present results, two hypotheses can be put forward to explain this particular neuropsychological phenotype. First, infection damages the CNS, through olfactory transmucosal invasion by the virus (direct pathway).^97^ Second, an excessive immunological reaction induces a cytokine storm^106^ or vessel inflammation, leading to cerebral vasculitis (indirect pathway).^107^ Whatever the cause, our results can also be attributed to the presence of a neurological vulnerability in some patients preceding infection with SARS-CoV-2. The latter may have a triggering and accelerating effect on this pre-existing neurodegenerative process,^108^ inducing a greater and more rapid cognitive decline than that usually observed in neurodegenerative pathologies. Recent studies seem to confirm that anosognosia for memory dysfunction is a feature of prodromal Alzheimer's disease, and is predictive of the conversion from mild cognitive impairment to Alzheimer's disease.^109^

From a clinical point of view, these results could be of great importance for patient management in the future. They suggest that several patients with COVID-19 who did not report any complaints had severe neuropsychological sequelae, associated with reduced brain connectivity. These could have an impact on their work and activities of daily living, and indicate the need to take account of anosognosia in studies of cohorts with post-COVID-19 syndrome, including not only patients with complaints, but also patients without complaints, in order to be able to quantify the prevalence of anosognosia and related disorders.

It is important to note that the present study had several limitations, starting with a possible recruitment bias. By enrolling volunteers, we may have selected the most severe cases in the mild group (who were interested in the study because of their cognitive complaints). Nevertheless, a large proportion of our patients had no complaints, either clinical or self-reported. Second, our anosognosic group had significantly more chronic renal failure, which may over the long term have had an influence on cognitive factors, although all the clinical variables were known and treated. Third, whereas there is a validated method for calculating self-appraisal discrepancy scores, there is no consensus on the calculation of anosognosia scores.^20^ Fourth, the statistical comparison of behavioural data and functional connectivity revealed an imbalance between the groups. Moreover, the small number of anosognosic participants who underwent MRI may limit the generalization of this group's neuroimaging data.

## Conclusion

To our knowledge, this study is the first to identify anosognosia for memory disorders as a factor that can be used to create subgroups of patients with cognitive/psychiatric post-COVID-19 condition symptoms. This anosognosia appears to reflect a broader dysfunction related to general self-awareness, possibly also concerning olfactory and respiratory symptoms, and involving brain networks that subtend self-monitoring. From a clinical point of view, this paves the way for further research, and also underlines the need to pay particular attention in the management of patients without complaints.

## Supplementary Material

fcac057_Supplementary_Data

## Abbreviations

aCompCor =: anatomical component-based noise correction
ANTs =: Advanced Normalization Tools
BRIEF-A =: Behavior Rating Inventory of Executive Functioning-Adult Version
CAM =: cognitive awareness model
CN_C_ =: control network C
CompCor =: component-based noise correction
DAN_B_ =: dorsal attention network B
DES =: Dissociative Experience Scale
DMN =: default mode network
DMN_B_ =: default mode network B
ECS =: Emotion Contagion Scale
EMIF-SEP =: Echelle Modifiée d’Impact de la Fatigue
ERQ =: Emotion Regulation Questionnaire
*f*MRI =: functional MRI
GERT =: Geneva Emotion Recognition Task
GREFEX =: Groupe de Réflexion sur l’Évaluation des Fonctions Exécutives
ISI =: Insomnia Severity Index
OFC =: orbitofrontal cortex
pCun =: precuneus
PFCl =: lateral prefrontal cortex
PCL-5 =: Posttraumatic Stress Disorder Checklist for DSM-5
PSS-14 =: Perceived Stress Scale—14 items
QPC =: Cognitive Complaints Questionnaire
SalVentAttn_A_ =: salient ventral attention network A
SF-36 =: Short Form 36 Health Survey
SomMot_A_ =: somatosensory motor network A
STAI-S =: State Anxiety Inventory
STAI-T =: Trait Anxiety Inventory
TAP =: Test of Attentional Performance
tCompCor =: temporal component-based noise correction
WAIS-IV =: Wechsler Adult Intelligence Scale—Fourth Version
WM =: white matter
