# Supplementary material for: Functional connectivity underlying cognitive and psychiatric symptoms in post-COVID-19 syndrome: is anosognosia a key determinant?
Source: Brain Commun. 2022 Mar 9;4(2):fcac057. doi: 10.1093/braincomms/fcac057 (PMC8956133; doi:10.1093/braincomms/fcac057)
Supplement: fcac057_Supplementary_Data [file fcac057_Supplementary_Data.docx]

**SUPPLEMENTARY INFORMATION**

**Supplementary Table 1. Sociodemographic data as a function of severity in the acute phase and awareness of memory dysfunction**

|  | Severity of acute infection | | | | Memory dysfunction awareness | | |
| --- | --- | --- | --- | --- | --- | --- | --- |
|  | Mild  (*n* = 19) | Moderate  (*n* = 21) | Severe  (*n* = 9) | K-W | Anosognosic  (*n* = 11) | Nosognosic  (*n* = 38) | M-W |
| Mean age (± *SD*) | 54.05 (± 8.77) | 55.00 (± 12.57) | 58.00 (± 11.87) | .716 | 49.92 (± 9.03) | 57.05 (± 11.04) | .061 |
| Mean education level (± *SD*) [Levels 1-3]^a^ | 2.68 (± 0.48) | 2.79 (± 0.42) | 2.73 (± 0.47) | .766 | 2.75 (± 0.45) | 2.73 (± 0.45) | .923 |
| Sex (F/M)  [% women] | 7/12 [36.84 %] | 8/13 [38.01 %] | 0/9 [0.00 %] | **.044*** | 4/7 [36.36 %] | 11/27 [28.94 %] | .861 |
| Handedness  (left-handed) | 1 | 0 | 0 | - | 0 | 1 | - |

*^Note.^* ^M-W: Mann‑Whitney^ *^U^* ^test; K-W: Kruskal‑Wallis ANOVA test.^

^*^ *^p^* ^< .050.^

^a^ Level 1 is equivalent to compulsory schooling in Switzerland (< 11 years of study), Level 2 is equivalent to a vocational diploma (11‑12 years of study), and Level 3 is equivalent to the Matura high-school diploma level and higher education (> 12 years of study).

**Supplementary Table 2. Intervals between infection and neuropsychological testing, between infection and MRI, and between neuropsychological testing and MRI, as a function of severity in the acute phase and awareness of memory dysfunction, for the subgroup of patients (*n* = 49) who underwent MRI.**

|  | Severity of acute infection | | | | Memory dysfunction awareness | | |
| --- | --- | --- | --- | --- | --- | --- | --- |
|  | Mild  (*n* = 19) | Moderate  (*n* = 21) | Severe  (*n* = 9) | K-W | Anosognosic  (*n* = 11) | Nosognosic  (*n* = 38) | M-W |
| Number of days between infection and neuropsychological testing (mean ± *SD*) | 225.00 (± 44.48) | 243.05 (± 55.63) | 230.18 (± 40.02) | .566 | 237.17 (± 39.78) | 231.86 (± 50.80) | .385 |
| Number of days between infection and MRI (mean ± *SD*) | 253.00 (± 41.75) | 281.42 (± 50.56) | 283.27 (± 51.94) | .076 | 287.08 (± 38.30) | 265.54 (± 51.07) | .146 |
| Number of days between neuropsychological testing and MRI (mean ±*SD*) | 28.00 (± 20.91) | 38.37 (± 26.28) | 53.09 (± 25.07) | **.032*** | 49.90 (± 26.95) | 33.68 (± 23.93) | .078 |

*^Note.^* ^M-W: Mann‑Whitney^ *^U^* ^test; MRI: magnetic resonance imaging; K-W: Kruskal‑Wallis ANOVA test.^

**Supplementary Table 3. Acquisition parameters for structural images (T1w MPRAGE).**

| Acquisition time | 4 min 36 s |
| --- | --- |
| Repetition time | 2300 ns |
| Echo time | 2.24 ms |
| Acquisition matrix | 256 x 256 |
| FOV | 186 x 239 x 239 mm |
| Flip angle | 8° |
| Number of slices | 208 (sagittal) |
| Voxel size | 0.9 x 0.9375 x 0.9375 mm |
| Slice thickness | 0.9 mm |

**Supplementary Table 4. Acquisition parameters for functional images (rs-fMRI).**

| Acquisition time | 7 min 59 s |
| --- | --- |
| Repetition time | 1 s |
| Echo time | 30 ms |
| Acquisition matrix | 88 x 88 |
| FOV | 220 x 220 x 160 mm |
| Flip angle | 50° |
| Number of slices | 64 (transversal) |
| Voxel size | 2.5 x 2.5 x 2.5 mm |
| Slice thickness | 2.5 mm |

**Supplementary Table 5. MRI motion data as a function of severity in the acute phase and awareness of memory dysfunction**

|  | Severity of acute infection | | | | Memory function awareness | | |
| --- | --- | --- | --- | --- | --- | --- | --- |
|  | Mild  (*n* = 19) | Moderate  (*n* = 21) | Severe  (*n* = 9) | K-W | Anosognosic  (*n* = 11) | Nosognosic  (*n* = 38) | M-W |
| Mean FD | 0.23 (± 0.11) | 0.28 (± 0.15) | 0.29 (± 0.09) | .326 | 0.23 (± 0.1) | 0.27 (± 0.13) | .508 |
| Excluded volumes | 8.15 (± 19.79) | 25.38 (± 61.94) | 18.11 (± 27.61) | .532 | 9.67 (± 19.62) | 19.86 (± 49.35) | .653 |

*^Note.^* ^FD: framewise displacement; M-W: Mann‑Whitney^ *^U^* ^test; K-W: Kruskal‑Wallis ANOVA test.^

**Supplementary Figure 1. Results of visual analysis of MR images and comparison between anosognosic and nosognosic patient groups.**


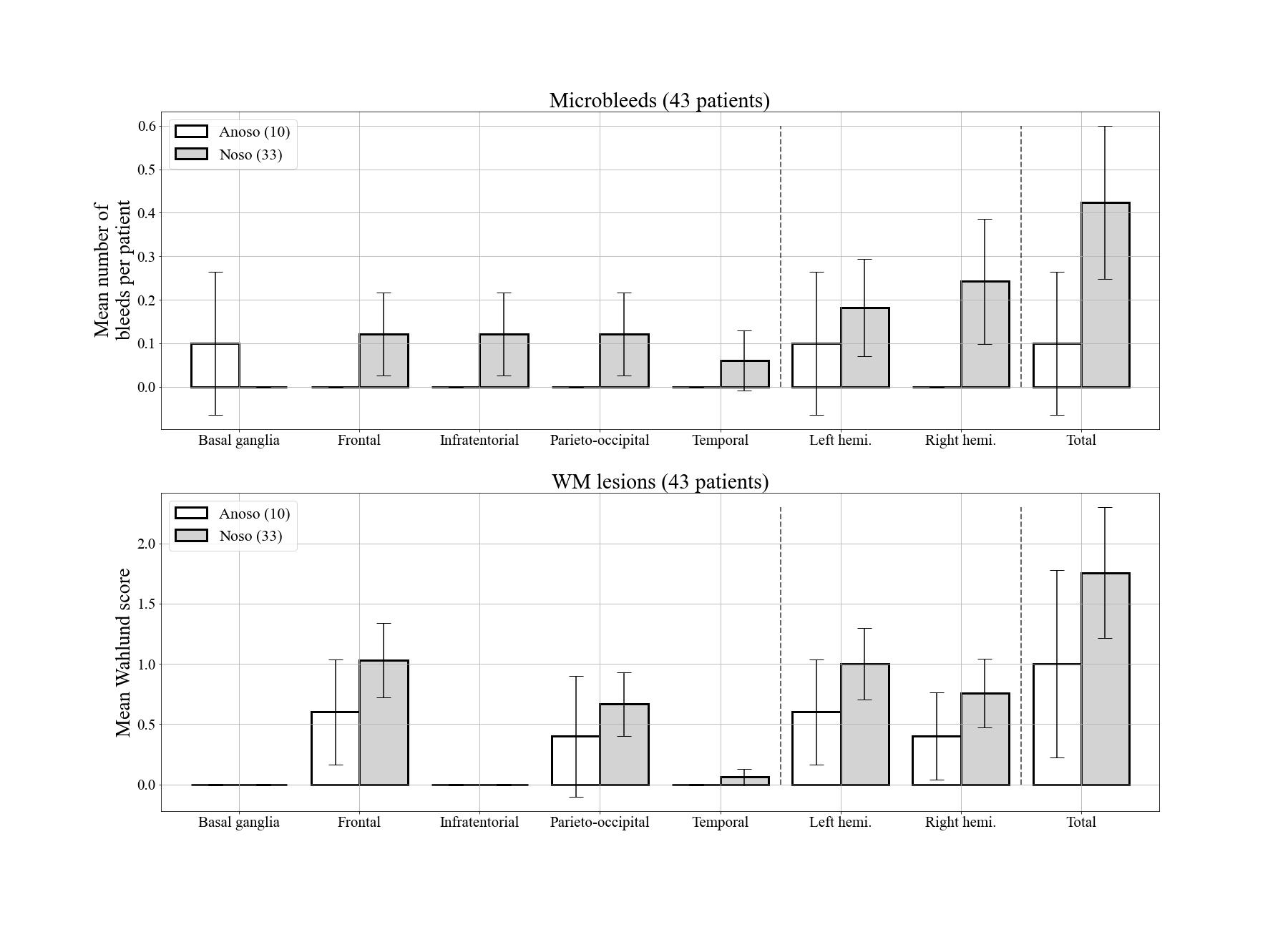


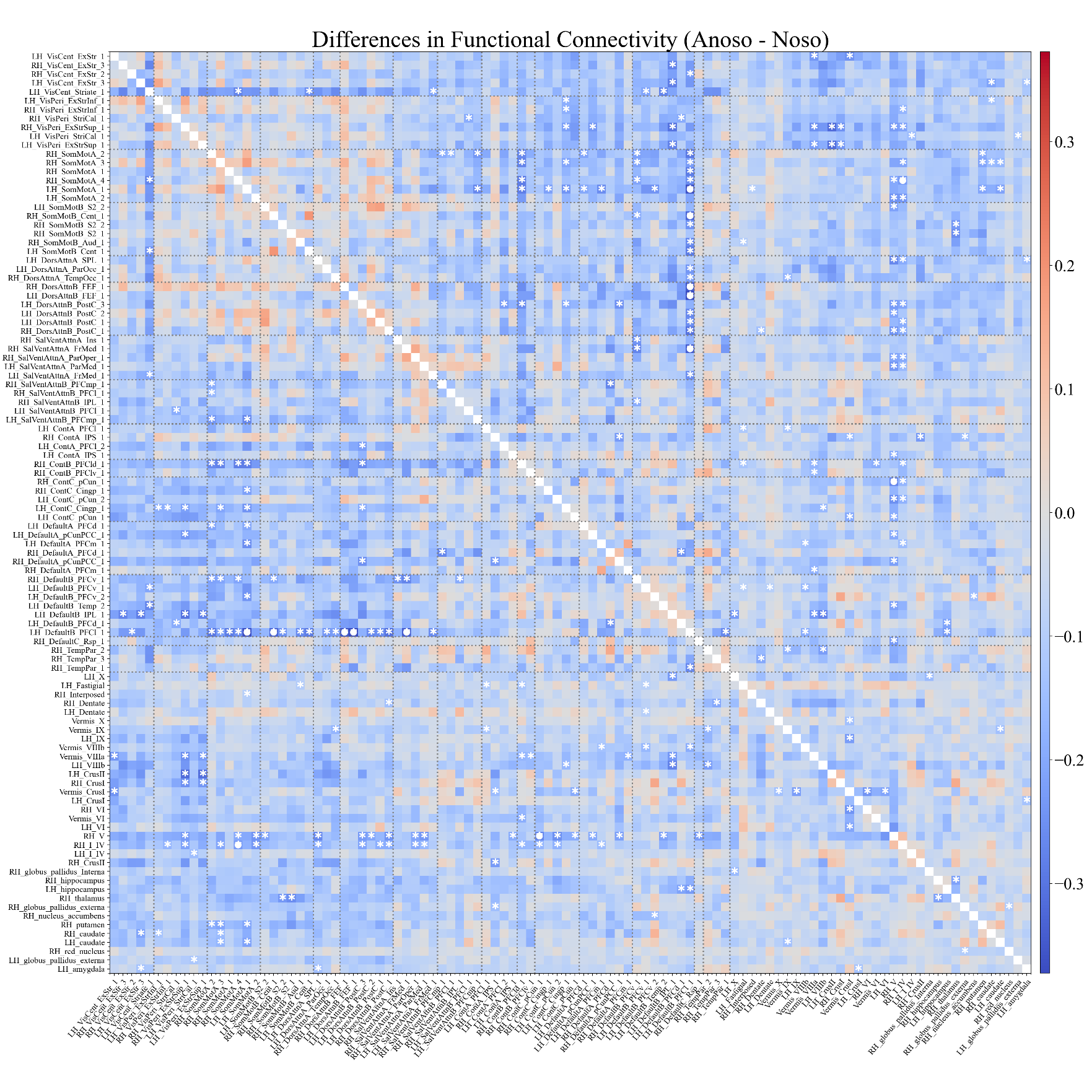
**Supplementary Figure 2. Hypoconnectivity trends in patients with anosognosia for memory dysfunction.**

- Uncorrected *p* < 0.01.
- FDR corrected (*p* < 0.05).
